# Estimating the magnitude and scope of disability-related direct costs: a systematised review

**DOI:** 10.1007/s10198-025-01851-x

**Published:** 2025-11-20

**Authors:** Lena Morgon Banks, Zachary Morris, Sara Rotenberg, Daniel Mont, Monica Pinilla-Roncancio, Ludovico Carraro, Alex Cote, Mercoledi Nasiir, Jill Hanass-Hancock, Stephen McGarity, Pamela Smith, Sophie Mitra

**Affiliations:** 1 International Centre for Evidence in Disability, London School of Hygiene & Tropical Medicine, Keppel St, London, WC1E 7HT United Kingdom; 2 Stony Brook University, New York, NY USA; 3 Center for Inclusive Policy, Washington, DC USA; 4 School of Medicine, Universidad de los Andes, Bogota, Colombia; 5 Independent Consultant, Oxford, UK; 6 UNICEF, New York, NY USA; 7 Prospera, Jakarta, Indonesia; 8 Gender and Health Research Unit, South African Medical Research Council, Durban, South Africa; 9 School of Health Science, University of KwaZulu-Natal, Durban, South Africa; 10 University of Tennessee, Knoxville, TN USA; 11 Sociedad y Discapacidad, Lima, Peru; 12 Fordham University, New York, NY USA

**Keywords:** Disability, Extra costs, Standard of living, Poverty, Out-of-pocket payments, Social protection

## Abstract

**Background:**

Globally, people with disabilities face a heightened risk of poverty, the extent of which is often underestimated due to a failure to account for disability-related direct costs or “extra costs”. People with disabilities spend significant proportions of their income on additional goods and services (e.g. personal assistance, assistive devices, extra healthcare, transportation): paying out-of-pocket for these items can substantially lower their standard of living, while inability to meet these costs can affect their well-being and social participation. Estimating the magnitude and sources of disability-related direct costs is key for policy and planning, as it can affect determinations of poverty and also identify where additional investment may be needed to reduce the individual burden of these costs (e.g., through social protection, designing more inclusive services).

**Objective:**

To synthesize evidence from recent studies on the magnitude and sources of extra costs amongst people with disabilities, comparing how estimates differ by methodological approaches, settings and individual characteristics.

**Methods:**

We conducted a systematised review of studies published between 2015 and January 2023. Searches covered 10 databases and the grey literature.

**Results:**

We retrieved 75 studies covering 67 countries, which reported substantial costs that often significantly increased the risk of poverty amongst people with disabilities. There was high heterogeneity across studies, depending on methodological approach and individual and contextual factors.

**Conclusions:**

These findings indicate a pressing need to offset individual spending on costs, such as through more extensive social protection and through designing more inclusive services (e.g., health, education, transport). There is also a need for more consensus on best practices for measuring extra costs across methodological approaches.

**Supplementary information:**

The online version contains supplementary material available at 10.1007/s10198-025-01851-x.

## Introduction

Globally, people with disabilities face a heightened risk of poverty [1–3]. This relationship between disability and poverty is increasingly recognised by the international community, including in social protection and other poverty alleviation activities [3]. For example, the 2030 Sustainable Development Agenda calls for a greater focus on disability across its 17 Sustainable Development Goals (SDGs), including SDG 1 which seeks to eliminate poverty “in all its forms” [4]. Disaggregation of SDG indicators by disability and other characteristics is an overarching principle of the Sustainable Development Agenda, so that “no one is left behind” from development gains [4]. The right of people with disabilities to an adequate standard of living is also codified in Article 28 of the United Nations Convention on the Rights of Persons with Disabilities (UNCRPD), which has been ratified by 183 States and the European Union [5].

However, indicators used in the SDGs and by governments for measuring poverty often fail to adequately capture economic well-being amongst people with disabilities and their households [6–8]. People with disabilities and their households often face disability-related costs that lower their standard of living. These costs include** indirect costs**, including fewer opportunities for work and education for both people with disabilities and members of their family (e.g., providers unpaid care and support). Indirect costs are reflected in many determinations of poverty (e.g., households have lower incomes and are therefore more likely to be below the national poverty line); however, most assessments assume equivalent consumptions needs between people with and without disabilities and thus fail to account for

 disability-related **direct costs**, or what is commonly referred to as “extra costs of disability” [9]. Disability-related direct costs are additional expenses that people with disabilities and their households incur to achieve a similar level of participation and well-being compared to people without disabilities [10]. They may include expenses for disability-related goods and services (e.g., rehabilitation, personal assistance, assistive devices) or additional spending on items also used by people without disabilities (e.g., general healthcare, transportation) [11]. The type and magnitude of disability-related direct costs varies amongst people with disabilities, depending on factors such as their impairment type, age and the environment in which they live [9, 12]. For example, the availability and affordability of required goods and services in different settings affects the out-of-pocket costs borne by individuals and their households. Further, public investment in disability-inclusive planning, such as inclusive education and healthcare, and accessible infrastructure, transportation and communication systems, can reduce the need for spending by households.

Estimating the magnitude and sources of disability-related direct costs is key for policy and planning, including for social protection programmes [13, 14]. Exploring the sources of direct costs - including unmet needs - can identify the types of goods and services required by people with disabilities and barriers to access, which can highlight priorities for public investment. Meanwhile, estimating the magnitude of costs can indicate the economic burden borne by individuals and their households, and affect determinations of poverty or benefit levels. For example, the Zambia Social Cash Transfer provides higher benefit levels to households with members with disabilities to account for direct costs [15].

Mitra et al. (2017) conducted a systematised review on disability-related direct costs, which covered the literature up to and including 2014. It retrieved 20 studies across 10 countries, the majority of which (*n*= 18) were from high-income settings [9]. Overall, costs were high, with substantial variability due to factors such as disability severity and household composition. Generally, costs increased with severity of disability and with smaller households.

There has been increasing attention to the importance of disability-related direct costs since the publication of this review, as well as further debate on the methods for quantifying disability-related direct costs. For example, the goods and services required approach was not widely used and its methodology has been advanced since the period covered by this review [16]. Consequently, this new systematised review seeks to update the review by Mitra et al. (2017), covering the literature from 2015 onwards. It compiles estimates of disability-related direct costs, both overall and by category of spending. It also compares estimates by methodological approach and across settings and participant characteristics such as age group and disability type. Finally, the review assesses the impact of these costs on poverty and explores the role of social protection in helping to mitigate costs.

### Approaches to measuring disability-related direct costs

Four main approaches have been used for quantifying direct costs [9, 13, 16–18].

### Goods and services used approach

The *goods and services (GS) approach* collects data on expenditures made by people with disabilities and their households that they deem to be linked to their disability. Data is used to estimate mean or median spending on direct costs within the sample. It can also be used to identify which items represent major sources of spending. The advantage of this approach is its simplicity, the focus on disability-related expenses and, depending on the sampling design, the possibility to provide separate estimates by type of disability.

A drawback of this approach is that it only captures actual spending, rather than the costs of all goods and services required for participation [13]. Given the link between disability and poverty [1], many households will not be able to afford all needed items due to financial constraints or choose not to allocate resources towards them (e.g., due to discrimination of disability within the household; beliefs that items won’t be useful). Further, in many settings, required items or services may have limited availability (e.g., lack of specialist healthcare, assistive products, inclusive education) or people may face additional barriers to purchasing them (e.g., lack of information, lack of autonomy in household financial decision-making) [3]. Further, the quality and utility of purchased items may be poor (e.g., visiting multiple health providers searching for appropriate services due to lack adequate information and referral mechanisms; assistive devices that are not properly fitted or appropriate to a person’s environment), and so it is unknown if their acquisition ultimately improves standard of living. Finally, respondents may find it difficult to disentangle what is additional spending due to disability, particularly on items also used by people without disabilities (e.g., healthcare, transport).

### Consumption patterns analysis

Similar to GS, consumption patterns analysis (CPA) also estimates spending on disability-related goods and services. CPA compares differences in expenditures between people with and without disabilities, particularly for categories where additional expenditures are likely (e.g., health, transport). CPA often involves secondary analysis of existing surveys with data on expenditures and disability status of household members, such as household income & expenditures surveys (HIES). The increasing uptake of questions on disability in household surveys, such as the Washington Group questions and other tools, has improved the availability of data to perform such analyses [19–21].

CPA carries many of the same limitations as GS. Nevertheless, comparing expenditures to households without members with disabilities can partially overcome one of the limitations of GS, in that it can help to disentangle what are “extra” expenditures for people with disabilities, particularly for goods and services also required by people without disabilities (e.g., health, transport). Focusing on households that have similar overall expenditures can show households’ divergent priorities in response to the needs of people with disabilities within the household. However, people with disabilities and their households often have lower incomes and other financial resources than households without members with disabilities [1], and so comparisons should factor in the economic resources available to households.

Another limitation of CPA is that analyses are limited by data availability. For example, standard HIES surveys often do not include questions on common items required by people with disabilities (e.g., personal assistance, assistive products), likely underestimating spending in these categories. Further, sample sizes of households with members with disabilities in secondary datasets may be underpowered for some analyses, such as exploring differences in consumption patterns by other characteristics (e.g., disability type, location).

### The standard of living approach

The *expenditure equivalence* or *standard of living (SOL) approach *estimates the direct disability-related costs incurred by using regression models to estimate the average additional income/consumption needed by a household with a member with a disability to have the same standard of living as households without members with disabilities [10, 22] This additional income is presumed to reflect out-of-pocket disability-related expenditures. The rationale being that households with members with disabilities are diverting some of their resources to disability-related direct costs, and so have a lower standard of living than would be expected for their level of wealth (e.g., income) and other characteristics of their household (e.g., size, location).

This approach has grown in popularity, as it can be calculated through secondary analysis of regularly collected data from socio-economic surveys (e.g., HIES) rather than requiring primary data collection [13]. While this approach only approximates actual spending, it considers disability-related expenditure in relation to a measure of living standards and so it adjusts for the lower income/consumption expenditures of people with disabilities.

However, the SOL approach has its limitations. Firstly, as with the previous methods, SOL does not capture unmet needs and thus will likely be an underestimate of the true value of needed goods and services particularly in settings where average household incomes are low and required items are in low availability. Second, this method on its own does not provide information on the types of goods and services people with disabilities are purchasing, which are important for informing policy responses. CPA can be used to complement SOL if using survey data that captures consumption expenditures, although as mentioned above, questions on spending on disability-related goods and services may not be adequately captured in surveys. Third, the original equation assumes a consistent, proportional relationship between spending on extra costs across a range of incomes or levels of consumption expenditures. This assumption can be tested using income or consumption expenditure in different functional forms. However, the functional form will reflect the relationship between standard of living and income/consumption expenditure and not the way such relationship changes because of disability. In reality, the proportion of household resources spent on extra costs may be highly variable by wealth, particularly in low-income settings: with high levels of poverty, many households may be able to meet only more basic needs until they have achieved a relatively high level of wealth.

### The goods and services required approach

The *goods and services required (GSR) *method, unlike the previous approaches, does attempt explicitly to account for unmet needs [14]. GSR currently uses a primarily qualitative approach, although quantitative approaches are being trialled [23]. It is used to produce a list of the full range of goods and services required by people with disabilities to achieve a certain level of participation or well-being. It also uses case studies to illustrate typical total costs that would be borne by individuals and their families if they acquired all required items through out-of-pocket payments or access to government programmes. These lists and case studies are created separately for different disability types and severities/level of support required, and sometimes by other characteristics (e.g., rural vs. urban, age group).

GSR approaches are grounded in a budget standards approach [24]. A budget standards approach considers what basket of goods and services are essential for achieving a certain standard of living and the income required to purchase those items. Determining baskets of goods and services and their costs can be achieved through different approaches, which are developed for needs assessments across the whole population [25]. The *consensual budget standards* approach involves members of the public (e.g., through focus group discussions) who agree on the basket of goods and services required. For example, focus groups of people with different types of disabilities and/or their caregivers – including a mix of people by location, age group, gender – can be used to generate lists of goods and services required due to disability and their costs. The *expert-driven budget standards approach* uses experts (e.g., service providers, social care workers, representatives from Organisations of Persons with Disabilities (OPDs), parent support groups) to identify and cost the relevant basket of disability-related goods and services required.

A combination of these two approaches may also be used. For example, an emerging strategy is for experts (e.g., service providers, social care workers, representatives from Organisations of Persons with Disabilities (OPDs), parent support groups) to generate an initial list of goods and services required by people with a particular type of disability, such as visual impairment [14, 16, 26]. Lists are separated by severity (e.g., people who are blind vs. people with more moderate vision impairment), and depending on the purpose of data collection, by other characteristics (e.g., children, working-age adults, older adults; rural vs. urban). These lists include at a minimum a description of the goods and services required for that group, their costs (what is borne by individuals and households and what is borne by the state through subsidies or free provision) and frequency of use. Details on prices may be supplemented through market research. Focus groups of people with disabilities for whom the list is relevant to then validate and update the expert lists. Here, for example, at least two focus groups should be used, one for people who are blind and one for people are partially sighted, ensuring representation by characteristics such as gender, location and age group. These two groups – experts and focus groups – can also generate indicative case studies that demonstrate the total costs that would be typical for each group if all required items could be purchased (i.e. here, for someone who is blind and someone who has moderate visual impairment), and illustrate how costs can vary based on different circumstances (e.g., children attending school, adults who are working; people in rural vs. urban areas).

GSR is a relatively new approach and is so far the only methodology that considers unmet needs for disability-related goods and services. The comprehensive lists of possible required goods and services, as well as indicative individual level costs, can be helpful for planning programmes to reduce affordability, availability and other barriers (e.g., social protection, investment in more inclusive health, transport, education and other systems). It is important to emphasize the GSR, in its qualitative form, cannot provide estimates of average costs borne by people with disabilities and their households, or indicate population-level needs for items. The method also is not without other limitations and may need further adaptations as it is applied in different settings.

Firstly, not all people with disabilities are aware of the types of goods and services they require, particularly if these items are not widely available in their setting [12]. Awareness may also be tied to individuals’ income level and education [27]. Ideally, GSR studies should select people with disabilities who are more knowledgeable about the range of possible goods and services that could support participation (e.g., associated with OPDs). Using expert groups can also help to offset these limitations and to triangulate findings from people with lived experience [14]. Second, applying prices for items in settings with limited availability of required goods and services (e.g., lacking supply chains for many assistive products, no or low availability of specialist health and social care services) can be a challenge. Third, some required services for participation, such as inclusive education, can only be covered at a societal rather individual level and so may not figure into estimated costs. Households may pay for additional tutoring or adapted materials to improve their child’s participation, but without government investment in inclusive education there will always be unmet needs. Fourth, the use of some items may vary by preferences and culture, such as the acceptability of providing personal assistance through mostly unpaid family members (and thus is an indirect cost) or through paid services (direct cost). Finally, defining a threshold for a certain level of participation and well-being can be subjective. The interpretation of what is required to achieve “full” or “equal” participation may differ amongst people based on their preferences, and their expectations based on what is common for others to do. Both may be influenced substantially by a person’s area of residence, socioeconomic status and other factors. One of the first step in these studies should be to define the level of participation that is expected and refer to it in the selection of goods and services throughout. Some studies, mainly from high income countries, have used a reference budget from the general population to indicate what people without disabilities would require to achieve a given level of participation [28–30]. This approach can help distinguish what is an extra disability-related cost rather than additional items that people without disabilities would also seek to achieve a level of participation or standard of living beyond the indicated threshold.

## Methods

The aim of this systematised review is to summarise the literature on disability-related direct costs from 2015 onwards. Specific objectives include:

- To compile estimates of direct disability-related costs, both overall and by category of spending (e.g., healthcare, personal assistance)
- To compare estimates of direct disability-related costs by methodological approach, study setting and individual characteristics (e.g., by age group, impairment type/severity)
- To explore the impact of direct disability-related costs on poverty and the mitigating role of social protection

This systematised review was conducted in line with practices outlined in the PRISMA statement [31], although it did not undertake all elements required to be classified as a systematic review (e.g., no risk of bias assessment undertaken).

### Search strategy and information sources

Ten databases relevant to health, economics and social science (Embase, APA PsychInfo, Econlit, Global Health, Ovid Medline All, Social Policy and Practice, ELDIS, Web of Science, Scopus, PubMed) were searched in July-August 2021 and again in January 2023. Grey literature was searched through Google/Google Scholar, and the reference lists of included studies were reviewed for additional sources. Experts working on disability and poverty were also consulted to identify publications in either the grey or academic literature.

Comprehensive search terms were developed based on previous reviews [1, 9, 32]. They included terms related to disability (including specific impairment types) and costs (e.g., terms for expenditures, specific methodologies for measuring disability-related direct costs) (see S1 file for a sample search string).

### Eligibility criteria

Studies were eligible for inclusion if they provided estimates of the additional direct costs of disability borne by individuals or their households. We included studies that used broad measures of disability (e.g., self-reported functioning, activity limitations) as well as studies on specific impairments or disabling health conditions (e.g., autism, Alzheimer’s disease, vision impairment).

Included studies could measures extra direct costs using any of the described methodologies (GS, GSR, SOL, CPA). For CPA, studies had to include a comparison group of people without disabilities and use some form of statistical analysis to compare costs between groups to be eligible for inclusion, rather than present raw totals only. CPA studies comparing healthcare costs between people with and without disabilities were excluded if they used health facility-based recruitment, as this would likely underestimate direct costs by selecting people without disabilities who were in worse health than the general population.

Studies were excluded if they only provided estimates of costs borne by the governments, insurance programmes or sources other than the individual and their household, or non-disaggregated estimates of total societal costs. Studies focusing only on indirect costs, or that did not disaggregate direct and indirect costs, were excluded.

Studies were eligible for inclusion if they were published between 2015 to January 2023, to capture data published since a previous systematised review on this subject [9]. Pre-prints and online first articles were eligible for inclusion if they were publicly available by January 2023. Case reports, reviews and abstracts without full texts were excluded. Studies in English, French or Spanish were able to be reviewed, although database searching was only conducted in English.

### Study selection, extraction and analysis

All articles were screened by title/abstract and then by full text by one reviewer (LMB). Extracted data from included studies was checked by a second reviewer (SR). Data extracted from the final selection of studies included:

- Study design, including methodology for assessing additional costs, including type of costs covered
- Population characteristics (e.g., type of disability, age)
- Estimates of disability-related costs, including point estimates (mean, median), range and as a percent of income
- Effect of costs on poverty, role of social protection in mitigating costs
- Differences in costs amongst people with disabilities (e.g., by severity, gender)

Results were described narratively, as meta-analysis was not possible given the wide range of populations and methods for measuring extra costs. Ranges and point estimates of costs were transformed into USD 2017 purchasing power parity (PPP) and adjusted for inflation. PPP conversion factors from the World Bank for 2017 [33] were applied against estimates in the local currency. Estimates that had already been converted to US dollars or another currency were first converted back to the local currency using author provided exchange rates, or the average annual exchange rate for the year of the study. All estimates were adjusted for inflation until 2017 using World Bank annual inflation rates by country [34]. The midpoint was used as the starting point for studies covering multiple years, unless the authors had already adjusted their estimates to a set year.

Groupings by region and income level are based on World Bank classifications [35].

## Results

**Fig. 1:**
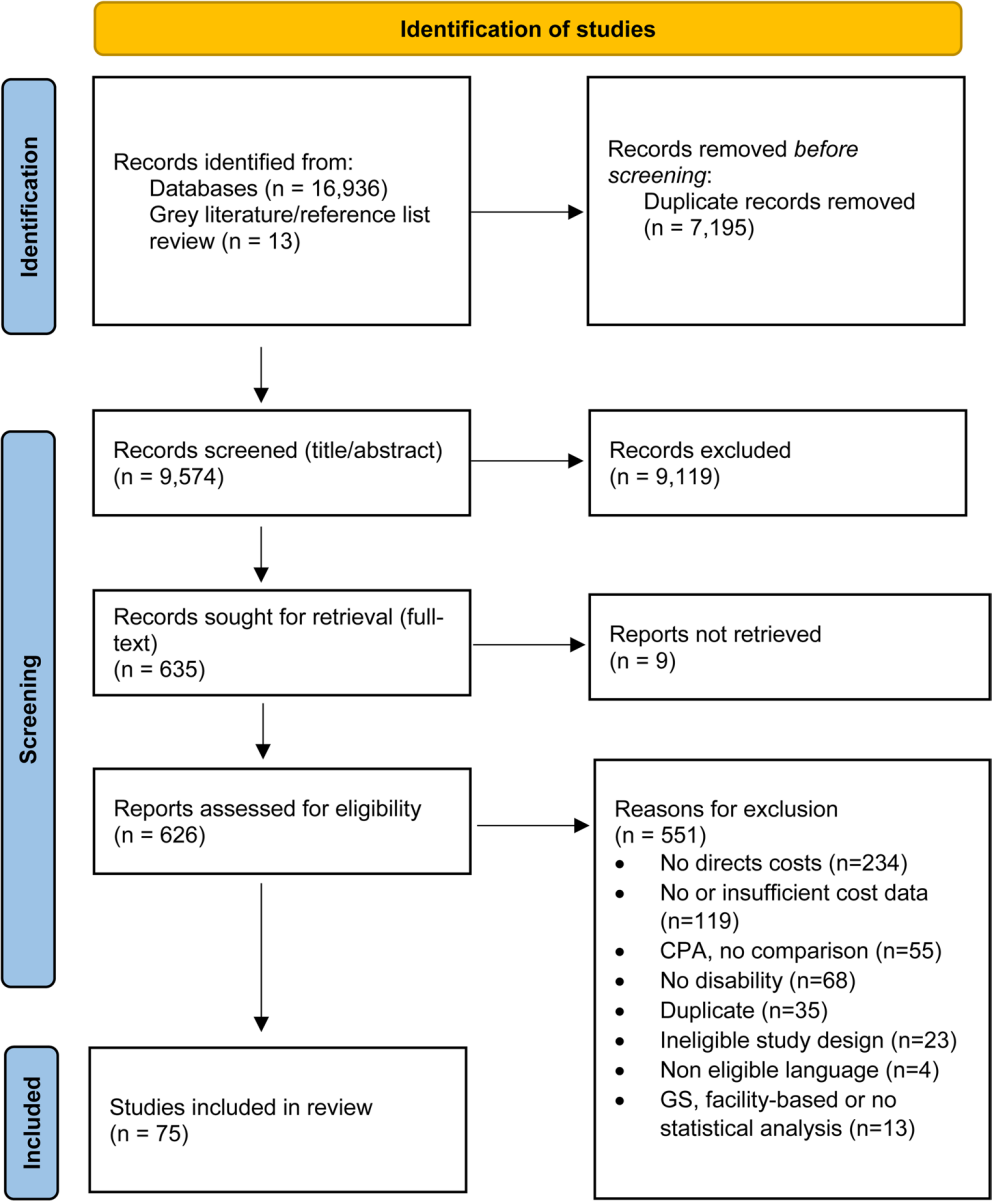
Flowchart of systematised review screening and identification

Database and grey literature/reference list review yielded a total of 16,946 records, of which 7,195 were duplicates. Of the 9,571 articles that were screened by title/abstract, 632 were assessed for eligibility by full text. In total, 75 studies met the study’s eligibility criteria (Fig. 1). Of these 75 studies, 41 (55%) focused on health-related costs.

These 75 studies covered 67 countries. The majority of studies were from high income countries (61%), with few studies from low-income countries (3%) (Table 1). By region, the majority of studies focused on East Asia and the Pacific (35%), North America (23%) and Europe and Central Asia (31%). By methodological approach, 39% used CPA, 32% used SOL, 21% GS and 4% used GSR. Amongst CPA studies, all covered healthcare costs only. Studies focused on a diverse range of age groups, with 19% on children, 31% on adults (any age), 15% on working age adults, 19% on older adults and 19% on all ages. By type of disability, most used broad measures or examined multiple impairment types (48%), followed by studies on cognitive/intellectual (19%), psychosocial (17%), physical (11%) and sensory (5%) limitations.

**Table 1:** Characteristics of included studies

Characteristic	Number of studies (%)
*Total*	75 (100%)
Region*	
East Asia and the Pacific	26 (35%)
Europe and Central Asia	23 (31%)
Latin America and the Caribbean	3 (4%)
North America	17 (23%)
Middle East & North Africa	1 (1%)
South Asia	2 (3%)
Sub-Saharan Africa	7 (9%)
Country income level*	
* Low-income*	2 (3%)
* Lower-middle income*	13 (17%)
* Upper-middle income*	18 (24%)
* High-income*	46 (61%)
*Age group* ^*1*^	
Children	14 (19%)
Adults (any age)	23 (31%)
Adults (working age only)	11 (15%)
Adults (older adults only)	14 (19%)
All ages	14 (19%)
*Disability type*	
Sensory	4 (5%)
Physical	8 (11%)
Intellectual/cognitive	14 (19%)
Psychosocial	13 (17%)
Mixed types/general measures	36 (48%)
- Washington Group questions	7 (9%)
Methodology*	
GS	16 (21%)
- Healthcare only	12 (16%)
CPA	29 (39%)
- Healthcare only	29 (39%)
SOL	24 (32%)
GSR	6 (8%)

*Total > 100% as some studies involved multiple settings

^*1*^Definition of age groups varied by study but typically was < 18 for children, 18–65 for working age and anywhere form 50 + for older age

### Estimates of total direct costs

A total of 28 studies covering 50 countries provided estimates of overall direct costs amongst people with disabilities (Table S1 & S2). Of these, 5 used GS, 23 used SOL, 5 used GSR and one used all three approaches. Twenty-six studies provided estimates in currency amounts (5 GS, 15 SOL, 5 GSR; 1 mixed types), while 25 provided estimates as a proportion of income (2 GS, 18 SOL, 4 GSR, 1 mixed).

The six studies using the GS approach only to estimate total direct disability-related expenditures were based in Malaysia, Canada, Ireland, Italy and South Africa (Table S2)[26, 36–39]. The mean annual direct costs incurred for children with cerebral palsy in Malaysia was estimated at $18,582 (68% of mean income) in one study and $24,954 (105% of mean income) in another [38, 39]. In Italy, costs incurred were $4,372 for adults with Alzheimer’s [37], while in Canada caregivers spent $2,468 on expenses for children with intellectual disabilities [36]. In South Africa, authors used an approach similar to GSR to explore direct costs amongst adults with a diverse range of disabilities [26]. The range of costs incurred amongst adults was highly heterogeneous depending on the type and severity of disability, from $57 for adults with mild psychosocial disability to more than $15,315 for people with severe physical disabilities per year. People with disabilities also reported high costs for unmet needs.

**Fig. 2:**
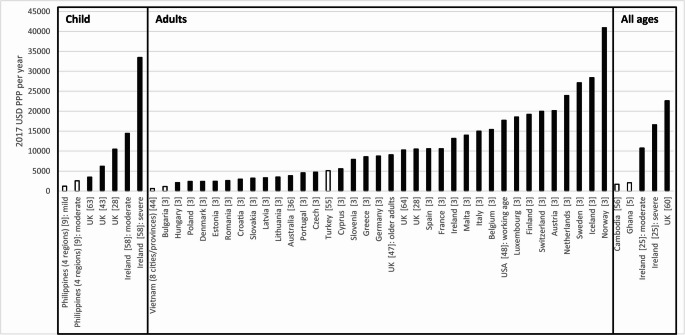
SOL estimates of direct costs in 2017 USD (PPP). Numbers in parentheses refer to citation number in File S2. Means were generated for studies using multiple models to estimate direct costs

**Fig. 3:**
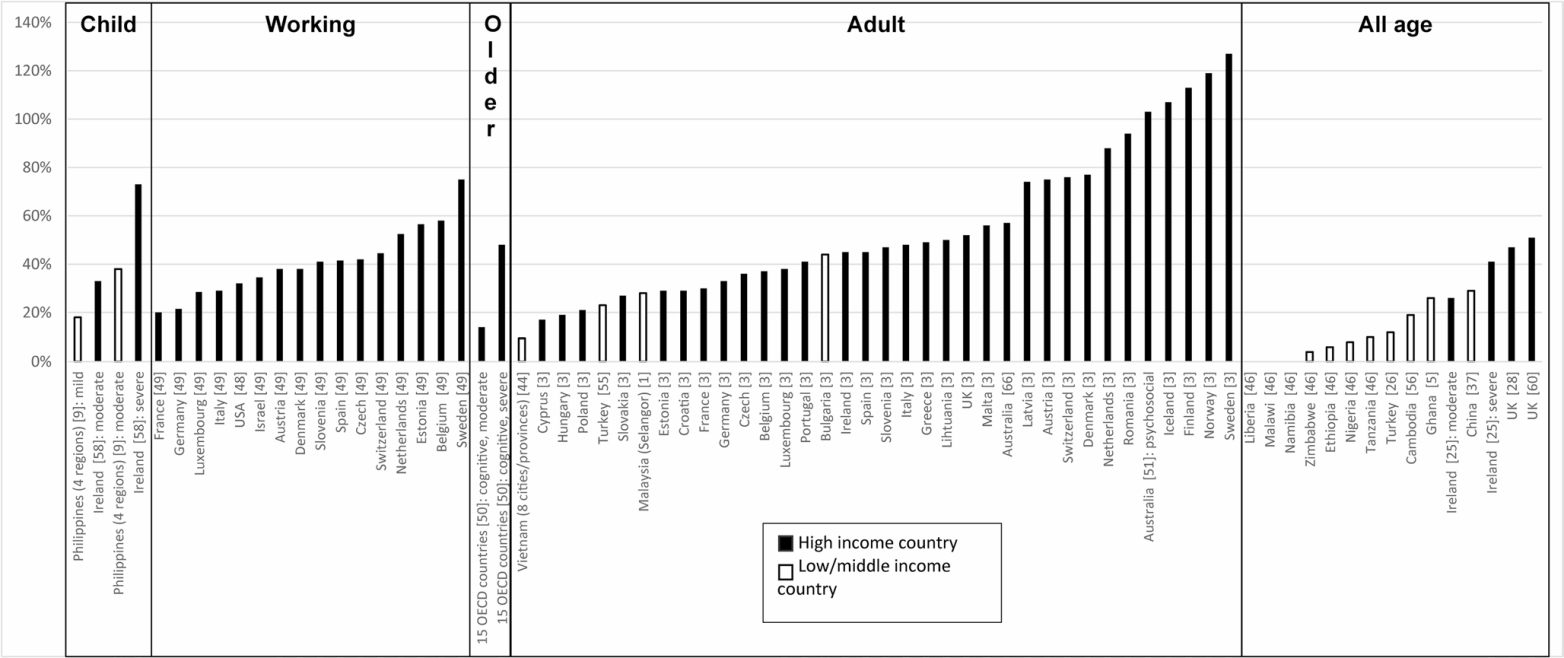
SOL estimates as a proportion of household income. Numbers in parentheses refer to citation number in File S2. Means were generated for studies using multiple models to estimate direct costs

The majority of studies used the SOL approach (*n* = 23) (Table S1). Figures 2 and 3 describes studies with estimates in US 2017 PPP and as a proportion of household income. Overall, SOL annual estimates of the direct costs made ranged from $604–658 for adults in Vietnam to $40,923 for adults in Norway, or between 14 and 155% of household income [40–42]. In low- and middle-income countriess, SOL estimates ranged from $604 for adults in Vietnam to $5,095 in Turkey, or between 4 and 28% of household income (with 3 non-significant estimates in Liberia, Malawi, and Namibia) [40, 42, 43]. Cost estimates using SOL were generally higher in high income settings, ranging from 14% of household income for older adults with moderate cognitive impairment in OECD countries to 155% of household income for adults with disabilities in Norway [41, 44]. As stated above these estimates do not necessarily reflect that required spending for full participation is more than in lower income settings, but that current spending is higher.

Meanwhile, six studies used the GSR, which were all in high-income countries (UK, New Zealand, Spain, Ireland) and mostly in urban settings (Table S2). In the UK, the estimated required costs ranged from $3,852-5,958 amongst adults with vision impairments and were $12,873 for working-age adults who were deaf, representing an increase of between 25 and 84% of an average budget for people without disabilities. In New Zealand, working-age people with physical impairments required between $24,393 (low/moderate) to $88,798 (severe) to meet disability-related extra costs [45]. In Spain, costs varied by disability type, ranging from $14,459 for people with hearing impairments who used sign language to $75,507 for working age adults with physical disability with high support needs [46].

Three GSR studies were from Ireland. One focused on adolescents with profound intellectual disabilities, finding that households required approximately $15,796 per year to achieve a minimally acceptable standard of living [28]. Another study from Ireland estimated that working-age adults with profound vision impairments required an additional $2,923 per year to achieve a minimally acceptable standard of living [29].

One study from Ireland compared total costs using all three methodologies [23]. The SOL approach estimated people with severe disability spent $14,860 − 18,269 per year (41% of household income) and people with moderate disability spent $9,778 − 11,684 per year (26% of household income). Meanwhile, GS estimates were on average $11,240 per year ($10,848 for moderate, $15,353 for severe). The study also estimated total required costs using a survey approach, which covered all people receiving a disability benefit nationally. The survey reported average required costs including unmet expenses were $14,611 per year ($14,418 for moderate, $20,277 for severe).

### Health-related expenditures

A total of 43 studies measured extra health-related costs, with 29 using CPA, 14 using GS and three using GSR (Fig. 2, Table S3). Of the 29 CPA studies comparing costs between people with and without disabilities or pre- and post-onset of disability, all but one found statistically significant higher healthcare costs amongst people with disabilities.

Overall, 19 studies were based in low-and-middle income countries. Annual costs were generally lower in low- and middle-income countries than in high income countries, with the lowest reported cost a median of $8 amongst people with physical impairments in Myanmar up to a mean of $11,408 for children with cerebral palsy in Malaysia [38, 47]. In high income countries, reported costs ranged from a minimum of an average of $45 per year for children with communication impairments in Australia, to a maximum of a mean of $4,406 for people with dementia in the US [48, 49]. Of the 24 studies set in high-income countries, 14 were from the US.

By type of disability, psychosocial disability represented the largest portion of studies (*n* = 12), followed by cognitive/intellectual impairments (*n* = 9). Eleven studies presented costs for multiple distinct types of disabilities. There were several studies reporting extra health-related costs in similar populations. For example, there were six studies from the US on extra health-related costs amongst older adults with cognitive impairment, ranging in estimates from $1,181 to $4,406 [49, 50]. Similarly, four studies in China explored the costs of psychosocial disabilities amongst adults, with costs ranging from $33 − 1,728 per year [51, 52]. Two studies explored costs of cerebral palsy in children in Malaysia, with mean costs ranging from $9,930 to $11,408 per year [38, 39].

Health-related direct costs could represent a large share of household resources, particularly in low- and middle-income countries. For example, healthcare costs for schizophrenia in Ghana were 31% of caregivers’ income, while in Vietnam healthcare costs reached as high as 31% of household income for people with severe functional limitations [53, 54]. In Nigeria, healthcare costs for children with cerebral palsy were greater than 79% of household income for 80% of the sample [55]. Three studies in the US measured extra as a percent of income, ranging from 3% for working age adults in multi-adult households to 16% in older adults with dementia [49, 56]. Costs for dementia care in the final seven years of life in the US decreased household wealth by 24% points compared to older adults dying of other causes [57]. Across 17 European countries, excess health costs represented 2% of household income [49].

**Fig. 4:**
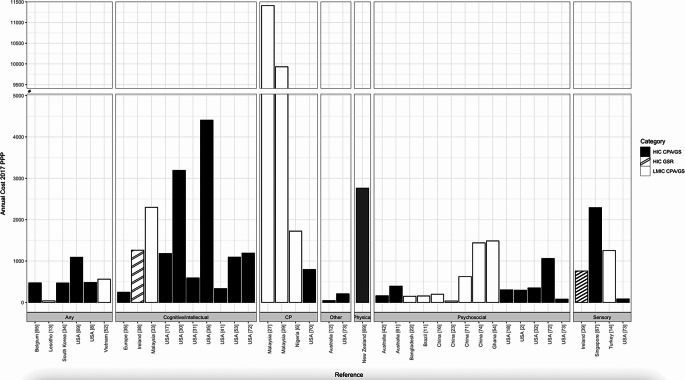
Annual mean extra health-related costs (2017 USD). Numbers in parentheses refer to citation number in File S2. CP refers to cerebral palsy

Five studies examined extra health costs as part of total direct costs: two studies in Malaysia on cerebral palsy in children found healthcare costs represented 28–53% of total costs [38, 39]; meanwhile in New Zealand, a GSR study found health costs represented 5% of total extra costs required for people with physical impairments [45]. In Ireland, GSR studies found extra costs for adolescents with profound intellectual disability were 8% of total extra costs required and 26% of total extra costs for working-age adults with vision impairments [28, 29] (Fig.4).

Two studies used longitudinal data to assess the impact of onset of disability on health expenditures. One study from Indonesia found the onset of activity limitations in adults in Indonesia was associated with a 31% increase in health expenditures [58]. In the US, onset of Alzheimer’s and related dementias resulted in excess health spending of $1,094 per year [59].

### Care and support

Personal assistance and other human support costs were assessed in eight studies (Table S4). Four studies used the GS approach. In Malaysia, two studies amongst children with cerebral palsy reported average spending for domestic helpers at $1,263 and $3,909, representing 7% and 17% respectively of total direct costs [38, 39]. In South Africa, costs for assistance ranged from $0 for people with moderate intellectual impairment up to $30,630 for people with deafblindness or who are deaf and accounted for up to 100% of total direct costs [26]. These costs varied depending on environmental and personal circumstances (e.g., people who were working, living in rural vs. urban areas). In Italy, private assistance was $4,245 per year, accounting for 97% of total direct costs [37].

Four studies used the GSR approach. In Spain, required costs for personal assistance/care staff ranged from $3,804 for people with moderate vision impairment to $39,910 for people with severe intellectual impairments; these costs represented between 21 and 78% of total direct costs estimates [46]. In New Zealand, required costs for support ranged between $20,233 − 61,720 for people with severe physical impairments, representing 70–83% of total direct costs [45]. In the UK, people who were deaf required $9,861 per year for interpreters (78% of total direct costs) [30]. In Ireland, care costs for adolescents with profound intellectual disability were $4,002 per year (25% of total costs) [28].

### Transport

Nine studies measured direct costs for transport (Table S5). Four studies used a GS approach for transport costs. In Malaysia, one study found spending averaged $1,805 per year for children with cerebral palsy, while in another transport costs were a median of $1,806 and a mean of $2,582 (10% of total direct costs in both studies) [38, 39]. In South Africa, spending on general travel ranged from $31 per year for people with psychosocial impairments to $7,535 for people with low functioning autism; people who were working often incurred additional travel costs, up to $2,840 for people with low-functioning autism [26]. In Canada, transport costs for parents of children with intellectual disabilities were a median of $476 per year (19% of total direct costs) [36].

Five used a GSR approach. In the UK, required transport costs for people with visual impairments ranged from $506-2,142 per year for adults with moderate to severe visual impairment (13–32% of total direct costs) and $423 for working-age adults who were deaf (3% of total direct costs) in urban settings [30]. In Barcelona, Spain, required costs for transport ranged $0 for some groups up to $15,421 for people with severe physical impairments (0–29% of total direct costs) [46]. In New Zealand, required transport costs were $5,446-7,034 annually for people with physical impairments (8–22% of total direct costs) [45]. In Ireland, working age adults with vision impairments living on public transportation routes required $866 less than a person without a disability due to the availability of discounted transportation [29]. Another study from Ireland found adolescents with profound intellectual disability required $4,762 more per year [28].

### Other sources of costs

One GS study assessed the cost of work-related supports, finding costs in working age adults with disabilities in the US ranged from $0–15,573 per year [60] (Table S6). Four studies explored direct costs related to education. In Malaysia, two GS studies of children with cerebral palsy reported costs between $326 to $939 per year, representing 2% and 4% respectively of direct costs [38, 39]. In Canada, a GS study reported the median cost for education and respite related expenses for children with intellectual impairments was $0, but up to $2,401 for children with severe/profound impairments [36]. In Ireland, a GSR study indicated a typical household with adolescents with profound intellectual disability would spend $763 per year less on education compared to a reference budget for a similar household without an adolescent with disabilities [28]. This difference was attributed to not paying for school books, stationary or State exam fees.

### Factors affecting direct costs

Overall, 31 studies explored differences in direct costs estimates amongst people with disabilities (Table S7).

Differences by severity were most frequently assessed (*n*= 20), with all finding costs increased with increasing severity of impairment or functional limitations [23, 30, 36, 40, 45, 46, 53, 61–73]. Nine studies explored differences by age group, with three studies finding no clear relationship with age [40, 46, 67], five finding costs higher for children/younger adults [23, 64, 74–76]and two reporting higher costs amongst older adults [30, 51]. Eight studies examined associations by location: three found higher costs in rural areas [51, 53, 77], one found higher costs in urban areas [75]; two found no clear difference by rural-urban [40, 76]; additionally, one study found spending at the country-level was roughly correlated with GDP [78], while another found higher costs were correlated to areas with larger disability employment gaps [70]. Nine studies assessed differences by household wealth/education, with three finding costs higher in richer households [23, 53, 58], five reporting higher costs in poorer households [51, 69–72]and one finding no difference by wealth [67]. Six studies explored difference by household structure, with three of four finding higher costs for single vs. multi-adult households [70, 79, 80]and three of three finding costs increased with more members with disabilities [67, 70, 75]. Six studies explored differences by gender, with three reporting higher costs amongst women [51, 81, 82], two reporting higher costs amongst men [64, 79]and one finding no difference [40]. Three studies disaggregated costs by employment status, with all finding slightly higher costs amongst people with disabilities who were out of work [67, 70, 80]. One from the US assessed differences by race, finding higher absolute spending amongst white respondents compared to other races [82].

### Impact of costs on poverty

**Fig. 5:**
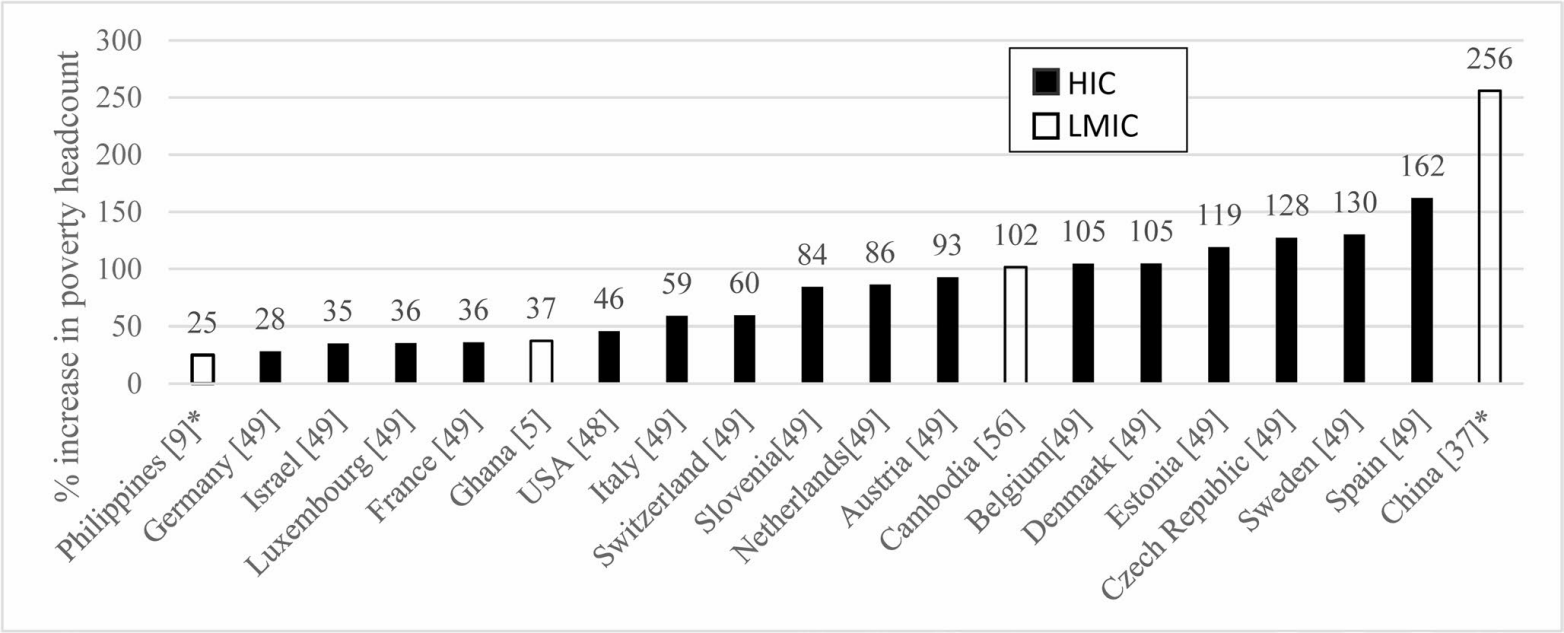
Percent increase in poverty headcount amongst households with members with disabilities due to direct costs. * Author calculated average of several estimates. Numbers in parentheses refer to citation number in File S2

Five SOL studies covering 20 countries explored the effect of direct costs on poverty headcounts (Fig. 5). Accounting for disability-related direct costs raised the proportion of households with people with disabilities living in poverty by 25% (Philippines), up to 256 pp (China). In 8 of the 20 countries, poverty headcounts amongst persons with disabilities’ households more than doubled after factoring in direct costs estimates.

### Role of social protection

Several studies explored the role of social protection in mitigating extra health-related costs. For example, two studies found health insurance could reduce direct costs related to healthcare amongst people with disabilities. In China, more generous health insurance schemes were tied to reduced excess out-of-pocket health costs for depression, as they offered a higher reimbursement rate [64]. Similarly, in the United States, extra health costs were lower for people with disabilities who had insurance. Still, in eight studies amongst insurance recipients, people with disabilities still had higher costs compared to people without disabilities. [48, 52, 64, 83–87]. The design of health insurance schemes could also affect the magnitude the costs. For example, some papers stated that schemes did not include adequate coverage for disability-related health services, particularly rehabilitation, specialist services and assistive devices [53, 84]. Further, high co-payments and deductibles could lead to excess health costs amongst insured people with disabilities [52, 84]. In turn, adaptations to health insurance schemes could reduce extra health-related costs amongst people with disabilities. For example, in the United States, a policy change in the reimbursement limits for mental health services was attributed with an annual decrease of US$149 (2017 PPP) in excess health costs for children with psychosocial impairments [84]. In Turkey, the implementation of a universal health insurance scheme was attributed to reducing all disability-related direct costs from 51% to 17% of household income [43].

Similarly, benefit levels from cash transfers were often insufficient to offset costs [29, 37, 65, 67, 69, 70, 88, 89]. For instance, in the United Kingdom, disability benefits represented less than 15% of spending on disability-related direct costs [70]. In Cambodia, the median amount of cash benefits received from the government represented 68% of direct costs; however, only 4% of people with disabilities received any form of cash transfer, and receipt was mostly concentrated amongst older adults with disabilities [88]. In Australia, the introduction of a new disability-targeted cash transfer with lower benefit levels was reported to have led to widening direct costs [65].

Finally, other policy changes could affect direct costs. Across 15 OECD countries, costs were lowest for adults with cognitive impairments in countries with generous public long-term care regimes [90].

### Methodological approaches

The three studies using GSR, which accounts for both met and unmet spending needs, tended to produce the highest estimates compared to studies with SOL or GS. For example, in Spain direct costs estimates using GSR ranged from US$14,459 − 75,507 (2017 PPP) [46], while SOL produced estimates of US$9,399 − 11,701 [41].

Even within studies using the same approach, different methodological decisions were taken that could affect the estimates of direct costs. For example, GSR studies used a different threshold for identifying required costs, with Hirsch & Hill (2016), MacMahon et al. (2022), MacMahon & Moloney (2017) focusing on goods and services required for a minimal acceptable standard of living, Wilkinson-Meyers et al. (2015) using an adequate standard of living and Puig & Segura (2021) assessing costs required for closer to equal participation. Indecon (2021) used a survey and asked people to identify the cost of items they felt they required but could not afford [23].

In SOL studies, estimates are highly influenced by the choice of the standard of living indicator. Included studies took different approaches to developing the standard of living indicators, including through asset indexes/counts (*n* = 10), self-assessment of financial well-being (*n* = 6), self-reported material deprivation (e.g., wanting but not being able to afford goods and services) (*n* = 12) and deprivation count on a multidimensional poverty index (*n*= 1). The variability of estimates produced with different standard of living indicators was highlighted in studies that ran alternative models of costs. For example, across 31 countries in Europe, the difference in direct cost estimates as a proportion of household income varied by 4 to 73% points depending on the standard of living indicator used [41]. Similarly, in the Philippines estimates of direct costs varied from 11 to 25% for mild functional limitations and 19–64% for moderate/severe functional limitations depending on the SOL indictor used (asset index produced the lowest estimates, while subjective assessment of well-being and deprivation count on a multidimensional poverty index produced similar estimates) [73].

Finally, estimates produced with GS and CPA approaches were dependent on source of recruitment. Population-based surveys could provide representative estimates of actual spending; however, these estimates might severely underestimate the true scope of direct costs, particularly in low- and middle-income countries where availability of required goods and services and capacity to pay for them is low. Alternatively, recruitment through service providers or Organisations of Persons with Disabilities likely produces non-representative estimates of actual spending amongst people with disabilities but may produce estimates closer to those obtained through GSR.

## Discussion

Overall, this systematised review found that disability-related direct costs were substantial, increasing the risk of poverty amongst people with disabilities and likely impeding many people’s access to needed goods and services. The magnitude of estimated direct costs was highly variable across and within studies, depending on context, disability-related factors (e.g., type and severity), individual characteristics (e.g., age, wealth) and methodological approach.

The review highlights the increased attention on estimating direct costs related to disability, with 75 studies in 67 countries published since the Mitra et al. (2017) review was completed. In the Mitra et al. (2017) review, 20 studies from 10 countries were retrieved from all preceding years and so this represents a significant expansion in both quantity and scope of the literature in less than a decade. Most studies used approaches that approximate current spending, with few (8%) using GSR to capture both met and unmet needs. The SOL approach in particular has been increasingly utilised, with 24 studies retrieved in this review compared to 5 in the original.

However, this review demonstrates that caution is needed in using and communicating SOL estimates – as well as GS and CPA. Estimates of total direct costs were typically lower in low- and middle-income countries compared to high income countries, likely reflecting greater unmet needs in the former. For example, in one analysis using SOL across seven sub-Saharan African countries, three countries had non-significant estimates of extra costs [91]. It is highly likely that people with disabilities responding in the surveys still had disability-related direct costs, but poor availability and low capacity to pay for items limited access to the extent that they were not reflected in SOL estimates. Further, SOL estimates were sensitive to design decisions, particularly the choice of the standard of living indicator [9]. For example, in the Philippines, extra costs for households with children with moderate/severe functional limitations were 19% when using an asset index and 64% with a multidimensional poverty deprivation count [73], while across 31 countries in Europe estimates increased by up to 217% when using a subjective measure of financial well-being compared to an asset index [78]. As these methods grow in popularity, it is critical that results are communicated appropriately to prevent misinterpretation: for example, that providing cash transfers equivalent to the value of a SOL estimate will be sufficient to cover all required expenditures, or on how to interpret large differences in SOL estimates when using different standard of living indicators.

At the time of this review, GSR studies still represented only a small - but growing - proportion of the literature, and all included studies were from high income settings (with 5 of 6 from Europe). Additional research using GSR in different settings is needed to understand the full extent of goods and services required. The GSR approach may need adapting particularly for low income settings, due to unavailability of many goods and services locally or even nationally [16]. Still, the exercise of producing a listing of required items can at least indicate where there is no or very limited availability of certain required items, to set priorities for state investment.

This review also highlights areas for strengthening social protection and other systems to address disability-related direct costs. Importantly, cash transfers are a critical tool, but alone they are unlikely to meet all required costs borne by households. Several studies in this review found that, for example, cash transfers and other entitlements were well below estimates of extra costs [37, 65, 67, 69, 70, 88]. Health-related costs were a large source of additional spending for many people with disabilities. Health costs were also highly variable, unpredictable and could require significant short-term spending (e.g., at onset of disability). As such, fixed value cash transfers are unlikely to be sufficiently flexible to meet the diverse range of health costs required by different individuals at different times. Social health protection (e.g., social health insurance, national health systems) can offer a more appropriate strategy for addressing disability-related health costs, in which individuals can access covered health services when needed with at least some level of financial protection [92]. Existing schemes may require adaptations, as some studies in this review and others found that many have limited coverage of impairment-related health services and products (e.g., assistive devices, rehabilitation, specialist services such as psychiatry, ophthalmology) in social health insurance [53, 93–95]. Policies such as subsidising user fees or capping out-of-pocket payments for people with disabilities, and covering costs such as transportation in seeking care, can also increase financial protection [84, 96].

Care and support and transport were other large sources of direct costs. These costs are likely to underestimated in included studies, as unpaid care and support by household members is common particularly in settings with limited formal care and support services [3]. Unpaid care and support, however, can carry substantial indirect costs [36, 37, 53, 86, 97–99]. Investing in care and support systems, including personal assistance, are possible strategies for mitigating these costs [100, 101]. For transportation costs, subsidies to public transportation and investment in accessible and widespread public transportation systems may help to offset these costs. For example, people with profound vision impairments in major cities in Ireland spent less on transportation than people without disabilities due to the availability of accessible public transport systems and disability-related concessions [29].

There was relatively little information about costs for accessing education and work. In many settings, people with disabilities face barriers that limit participation in work and education [101], and so actual spending in these categories can be lower than for people without disabilities. However, no or less spending compared to people without disabilities in these categories does not mean there are no required costs. For example, when required spending for employment (e.g., transport, reasonable accommodations covered by individuals) is particularly high, it may serve as a barrier to work.

Several limitations should be considered when assessing the findings of this review. Importantly, while some non-English articles were eligible for inclusion, the search string only included English search terms, likely excluding studies without at least an English title or abstract. Further, the majority of studies retrieved used the SOL, CPA or GS approaches. These approaches do not account for unmet needs for goods and services [9].

While an increasing number of studies have measured disability-related direct costs, further research is still required. First, more research is needed using the GSR approach to estimate the full value of required goods and services, particularly in low- and middle-income settings, given the lack of studies in these contexts. Second, studies comparing estimates generated across methods could help to illustrate gaps between met and unmet needs and highlight the compatibility of estimates generated by different approaches. Third, few studies used longitudinal data to track changes in costs over time. Longitudinal data can also be used to estimate costs from disability onset, which can reduce bias. However, only two study used longitudinal data to assess the impact of onset of disability [58, 64]. Finally, studies assessing the impact of social protection, inclusive services and other policies in improving capacity to pay for costs and reducing out-of-pocket spending are important to inform the policy decisions.

## Conclusion

This review illustrates a rapidly growing literature focused on measuring disability-related direct costs. Most studies used methods that approximate actual spending, rather than capturing all required goods and services. However, even these estimates indicate significant financial impacts on people with disabilities and their families, including sharp increases in the proportion of people living in poverty. Further work is needed to refine methodology across common approaches, and in communicating results, particularly in settings and amongst groups where unmet needs for required goods and services are likely to be high.

## Supplementary Information

Below is the link to the electronic supplementary material.

Supplementary Material 1 (DOCX 16.1 KB)

Supplementary Material 2 (DOCX 22.8 KB)

Supplementary Material 3 (XLSX 60.1 KB)

## Data Availability

All data is in the public sphere.
