# Supplementary material for: Estimating the magnitude and scope of disability-related direct costs: a systematised review
Source: Eur J Health Econ. 2025 Nov 20;27(5):969–85. doi: 10.1007/s10198-025-01851-x (PMC13451318; doi:10.1007/s10198-025-01851-x)
Supplement: Supplementary file 1 [file 10198_2025_1851_MOESM1_ESM.docx]

|  | **Embase, APA PsychInfo, Econlit, Global Health, Ovid Medline(R) All, Social Policy and Practice (searched Feb 3)** |
| --- | --- |
| **#** | **Searches** |
| 1 | ((disabilit* or disable* or handicap* or function* limitation* or function* diversit* or dependen* or special need*) adj6 (person* or people or individ* or patient* or subject* or household* or member*)).ti,ab. |
| 2 | (Physical* adj5 (impair* or deficienc* or disable* or disabili* or handicap* or incapacit*)).ti,ab. |
| 3 | (Cerebral pals* or Spina bifida or Muscular dystroph* or Arthriti* or Osteogenesis imperfecta or Musculoskeletal abnormalit* or Musculo-skeletal abnormalit* or Muscular abnormalit* or Skeletal abnormalit* or Limb abnormalit* or Amputation* or Clubfoot or Poliomyeliti* or Paraplegi* or Paralys* or Paralyz* or Hemiplegi* or wheelchair user* or wheel chair user*).ti,ab. |
| 4 | exp wheelchair user/ |
| 5 | ((Hearing or Acoustic or Ear or Ears) adj5 (loss* or impair* or deficienc* or disable* or disabili* or handicap*)).ti,ab. |
| 6 | ((Visual* or Vision or Eye or eyes) adj5 (loss* or impair* or deficienc* or disable* or disabili* or handicap*)).ti,ab. |
| 7 | (Deaf* or Blind*).ti,ab. |
| 8 | exp Hearing impairment/ |
| 9 | exp vision disorders/ |
| 10 | (Schizophreni* or Psychos#s or Psychotic Disorder* or Schizoaffective Disorder* or Schizophreniform Disorder* or Dementia* or Alzheimer* or anxiet* disorder* or depression* or Bipolar Disorder* or personality disorder*).ti,ab. |
| 11 | exp "schizophrenia and disorders with psychotic features"/ |
| 12 | exp Dementia/ or exp Alzheimer disease/ |
| 13 | ((Intellectual* or Mental* or Psychological* or Developmental or cognitive) adj5 (impair* or retard* or deficienc* or disable* or disabili* or handicap* or ill* or dysfunction* or deficit* or incapacit*)).ti,ab. |
| 14 | exp Mentally Disabled Persons/ |
| 15 | ((communication or language or speech or learning) adj5 (disorder* or disabilit* or impair* or deficit* or deficienc*)).ti,ab. |
| 16 | exp Learning Disorders/ |
| 17 | ((child* or juvenile) adj3 (disable* or handicap* or disabili*)).ti,ab. |
| 18 | exp Disabled Children/ |
| 19 | Exp disabled person/ |
| **20** | or/1-19 |
| 21 | ((Expenditure* or expense* or spend* or consumption) adj3 (health* or medic* or transport* or assist* or education or school or (activit* adj3 daily))).ti,ab |
| 22 | ((direct or extra or added or additional or excess or out-of-pocket or out of pocket) adj3 (cost* or expenditure* or expense* or spend* or consumption)).ti,ab |
| 23 | ((standard of living) adj3 (approach or method* or analys*)).ti,ab. |
| 24 | ((goods and services) adj3 (used or need* or requir*)).ti,ab. |
| 25 | (expenditure equivalen*).ti,ab. |
| 26 | ~~Exp healthcare cost/~~ |
| **27** | or/20-26 |
| **28** | 19 AND 27 (23308) |
| 29 | Limit 29 to yr="2015-Current" (3275) |
| 30 | Deduplicate (3175) |
|  |  |
