# Supplementary material for: Estimating the magnitude and scope of disability-related direct costs: a systematised review
Source: Eur J Health Econ. 2025 Nov 20;27(5):969–85. doi: 10.1007/s10198-025-01851-x (PMC13451318; doi:10.1007/s10198-025-01851-x)
Supplement: Supplementary file 2 [file 10198_2025_1851_MOESM2_ESM.docx]

1.	Amin, R.M. and N.S.M. Adros, *The Extra Costs of Having a Disability: The Case of IIUM.* Intellectual Discourse, 2019. **27**: p. 829-854.

2.	Ammerman, R.T., et al., *Annual direct health care expenditures and employee absenteeism costs in high-risk, low-income mothers with major depression.* Journal of Affective Disorders, 2016. **190**: p. 386-394.

3.	Antón, J.I., F.J. Braña, and R. Muñoz de Bustillo, *An analysis of the cost of disability across Europe using the standard of living approach.* SERIEs, 2016. **7**(3): p. 281-306.

4.	Arora, S., et al., *Societal cost of childhood intellectual disability in Australia.* Journal of Intellectual Disability Research, 2020. **64**(7): p. 524-537.

5.	Asuman, D., C.G. Ackah, and F. Agyire-Tettey, *Disability and Household Welfare in Ghana: Costs and Correlates.* Journal of Family and Economic Issues, 2020.

6.	Badaru, U.M., et al., *Analysis of Direct Monthly Cost of Outpatient Hospital-Based Care for Children With Cerebral Palsy in Kano, Nigeria.* Value in Health Regional Issues, 2019. **19**: p. 145-150.

7.	Banks, L.M., et al., *Disability and the achievement of Universal Health Coverage in the Maldives.* Plos one, 2022. **17**(12): p. e0278292.

8.	Bernard, D., T. Selden, and S. Yeh, *Financial burdens and barriers to care among nonelderly adults: The role of functional limitations and chronic conditions.* Disability and Health Journal, 2016. **9**(2): p. 256-264.

9.	Carraro, L., A. Robinson, and B. Hakeem, *Cost of raising children with disabilities in the Philippines*. 2022, UNICEF Philippines: Manila.

10.	Chiatti, C., et al., *The economic impact of moderate stage Alzheimer's disease in Italy: evidence from the UP-TECH randomized trial.* International Psychogeriatrics, 2015. **27**(9): p. 1563-1572.

11.	Chiavegatto Filho, A.D.P., et al., *Incremental health expenditure and lost days of normal activity for individuals with mental disorders: results from the São Paulo Megacity Study.* BMC Public Health, 2015. **15**(1).

12.	Cronin, P., et al., *The impact of childhood language difficulties on healthcare costs from 4 to 13 years: Australian longitudinal study.* International Journal of Speech-Language Pathology, 2017. **19**(4): p. 381-391.

13.	de Groot, R., et al., *The impact of the Lesotho Child Grant Programme in the lives of children and adults with disabilities: Disaggregated analysis of a community randomized controlled trial.* International Social Security Review, 2021. **74**(2): p. 55-81.

14.	Deniz, B., et al., *Direct Health Expenditure Analysis Related to Hearing Loss in Individuals Using Hearing Aids and Cochlear Implants.* Turkish Archives of Otorhinolaryngology, 2022. **60**(3): p. 142.

15.	Denny-Brown, N., B. O’Day, and S. McLeod, *Staying Employed: Services and Supports for Workers With Disabilities.* Journal of Disability Policy Studies, 2015. **26**(2): p. 124-131.

16.	Ding, R., et al., *Medical service utilisation and direct medical cost of depression: a cross-sectional analysis of urban medical claims data from China.* BMJ open, 2022. **12**(9): p. e056422.

17.	Dwibedi, N., et al., *Alzheimer Disease and Related Disorders and Out-of-Pocket Health Care Spending and Burden among Elderly Medicare Beneficiaries.* Medical Care, 2018. **56**(3): p. 240-246.

18.	Eisenberg, M.D., et al., *Health Care Spending by Enrollees With Substance Use and Mental Health Disorders in High-Deductible Health Plans vs Traditional Plans.* JAMA Psychiatry, 2020. **77**(8): p. 872-875.

19.	Genereaux, D., C.D. van Karnebeek, and P.H. Birch, *Costs of caring for children with an intellectual developmental disorder.* Disability & Health Journal, 2015. **8**(4): p. 646-51.

20.	Hanass-Hancock, J. and N. Deghaye, *Elements of the Financial and Economic Costs of Disability to Households in South Africa: A Pilot Study.* Department of Social Development. Pretoria, 2016.

21.	Hirsch, D. and K. Hill, *The additional cost of disability: A new measure and its application to sensory impairment.* Disability & Society, 2016. **31**(7): p. 897-913.

22.	Hossain, S.J., et al., *Prevalence of Maternal Postpartum Depression, Health-Seeking Behavior and Out of Pocket Payment for Physical Illness and Cost Coping Mechanism of the Poor Families in Bangladesh: A Rural Community-Based Study.* International Journal of Environmental Research & Public Health [Electronic Resource], 2020. **17**(13): p. 01.

23.	Hsieh, C.R. and X.Z. Qin, *Depression hurts, depression costs: The medical spending attributable to depression and depressive symptoms in China.* Health Economics, 2016. **27**(3): p. 525-544.

24.	Hu, Y., et al., *How does the onset of physical disability or dementia in older adults affect economic wellbeing and co-payments for health care? the impact of gender.* BMC Health Services Research, 2022. **22**(1): p. 701.

25.	Indecon, *The cost of disability in Ireland*. 2021, Department of Social Protection: Dublin.

26.	Ipek, E., *The Costs of Disability in Turkey.* Journal of Family and Economic Issues, 2019. **41**(2): p. 229-237.

27.	Ismail, A., et al., *The economic burden and determinant factors of parents/caregivers of children with cerebral palsy in Malaysia: a mixed methods study.* International Journal of Environmental Research and Public Health, 2022. **19**(1): p. 475.

28.	John, E., et al., *Disability Price Tag 2019.* Policy Report, 2019.

29.	Kamaralzaman, S., et al., *The economic burden of families of children with cerebral palsy in Malaysia.* Malaysian Journal of Public Health Medicine, 2018. **2018**: p. 156-165.

30.	Kelley, A.S., et al., *Residential setting and the cumulative financial burden of dementia in the 7 years before death.* Journal of the American Geriatrics Society, 2020. **68**(6): p. 1319-1324.

31.	Kennedy, J., E.G. Wood, and L. Frieden, *Disparities in Insurance Coverage, Health Services Use, and Access Following Implementation of the Affordable Care Act: A Comparison of Disabled and Nondisabled Working-Age Adults.* Inquiry, 2017. **54**: p. 46958017734031.

32.	Kennedy-Hendricks, A., et al., *Federal parity and spending for mental illness.* Pediatrics, 2018. **142**(2): p. 1-8.

33.	Kongpakwattana, K., et al., *A real-world evidence analysis of associations among costs, quality of life, and disease-severity indicators of Alzheimer's disease in Thailand.* Value in Health, 2019. **22**(10): p. 1137-1145.

34.	Lee, J.E., et al., *Catastrophic Health Expenditures for Households with Disabled Members: Evidence from the Korean Health Panel.* Journal of Korean Medical Science, 2016. **31**(3): p. 336-44.

35.	Lenzen, S., et al., *Health Care Use and Out-of-pocket Spending by Persons With Dementia Differ Between Europe and the United States.* Medical Care, 2021. **59**(6): p. 543-549.

36.	Li, J., et al., *Inequalities in standards of living: evidence for improved income support for people with disability.* 2019.

37.	Liao, J., et al., *Urban–rural difference in the costs of disability and its effects on poverty among people with disabilities in China.* Frontiers in Public Health, 2022. **10**: p. 989540.

38.	MacMahon, B., H. Boylan, and R. Thornton, *Care at Home: Costs of Care Arising from Disability*. 2022, Minimum Essential Budget Standards Research Centre & Family Carers Ireland.

39.	MacMahon, B. and N. Moloney, *A minimum essential standard of living for a single adult with vision impairment*. 2017, Minimum Essential Budget Standard Research Centre, National Council for the Blind of Ireland, Vincentian Partnership for Social Justice: Dublin.

40.	Mactaggart, I., et al., *A case-control study of musculoskeletal impairment: association with socio-economic status, time use and quality of life in post-conflict Myanmar.* BMC Public Health, 2019. **19**(1): p. 1502.

41.	Martinez-Martin, P., et al., *The long-term direct and indirect economic burden among Parkinson's disease caregivers in the United States.* Movement Disorders, 2019. **34**(2): p. 236-245.

42.	McIntyre, E., et al., *Conventional and complementary health care use and out-of-pocket expenses among Australians with a self-reported mental health diagnosis: a cross-sectional survey.* BMC health services research, 2021. **21**: p. 1-19.

43.	Melnychuk, M., F. Solmi, and S. Morris, *Using compensating variation to measure the costs of child disability in the UK.* The European Journal of Health Economics, 2018. **19**(3): p. 419-433.

44.	Minh, H.V., et al., *Estimating the extra cost of living with disability in Vietnam.* Global Public Health, 2015. **10**: p. S70-S79.

45.	Mitra, S. and J. Yap, *The Disability Data Report. Disability Data Initiative*. 2022, Fordham Research Consortium on Disability: New York.

46.	Mont, D., et al., *Estimating Households’ Expenditures on Disability in Africa: The Uses and Limitations of the Standard of Living Method.* International Journal of Environmental Research and Public Health, 2022. **19**(23): p. 16069.

47.	Morciano, M., R. Hancock, and S. Pudney, *Disability Costs and Equivalence Scales in the Older Population in Great Britain.* Review of Income and Wealth, 2015. **61**(3): p. 494-514.

48.	Morris, Z.A., et al., *The Extra Costs Associated With Living With a Disability in the United States.* Journal of Disability Policy Studies, 2020: p. 10442073211043521.

49.	Morris, Z.A. and A. Zaidi, *Estimating the extra costs of disability in European countries: Implications for poverty measurement and disability-related decommodification.* Journal of European Social Policy, 2020. **30**(3): p. 339-354.

50.	Morris, Z.A., A. Zaidi, and S. McGarity, *The extra costs associated with a cognitive impairment: Estimates from 15 OECD countries.* European Journal of Public Health, 2022. **31**(3): p. 647-652.

51.	Nghiem, S., et al., *Implicitly Estimating the Cost of Mental Illness in Australia: A Standard-of-Living Approach.* Applied Health Economics and Health Policy, 2020. **18**(2): p. 261-270.

52.	Nguyen, L., et al., *Health Service Utilization and Out-of-Pocket Expenditure Associated with the Continuum of Disability in Vietnam.* International Journal of Environmental Research & Public Health [Electronic Resource], 2021. **18**(11): p. 25.

53.	Oney, M., L. White, and N.B. Coe, *Out‐of‐pocket costs attributable to dementia: A longitudinal analysis.* Journal of the American Geriatrics Society, 2022. **70**(5): p. 1538-1545.

54.	Opoku-Boateng, Y.N., et al., *Economic cost and quality of life of family caregivers of schizophrenic patients attending psychiatric hospitals in Ghana.* BMC Health Services Research, 2017. **17**: p. 697.

55.	Ozdamar, O., E. Giovanis, and S. Samuk, *Household disability costs and living standards in Turkey: evidence from a health reform.* International Journal of Social Economics, 2020. **48**(2): p. 318-333.

56.	Palmer, M., J. Williams, and B. McPake, *Standard of Living and Disability in Cambodia.* Journal of Development Studies, 2019. **55**(11): p. 2382-2402.

57.	Puig, J. and M. Segura, *Economic comparative grievance of people with disabilites in the city of Barcelona.* Revista Espanola De Discapacidad-Redis, 2021. **9**(1): p. 153-178.

58.	Roddy, Á., *Income and conversion handicaps: estimating the impact of child chronic illness/disability on family income and the extra cost of child chronic illness/child disability in Ireland using a standard of living approach.* The European Journal of Health Economics, 2022. **23**(3): p. 467-483.

59.	Salinas-Rodríguez, A., et al., *Out-of-pocket healthcare expenditures in dependent older adults: Results from an economic evaluation study in Mexico.* Frontiers in Public Health, 2020. **8**.

60.	Schuelke, L., L. Munford, and M. Morciano, *Estimating the additional costs of living with a disability in the United Kingdom between 2013 and 2016.* The European Journal of Health Economics, 2022. **23**(2): p. 313-327.

61.	Sibbritt, D., et al., *Integrative health services use for depression in middle-aged and older Australian women.* European journal of integrative medicine, 2021. **46**: p. 101367.

62.	Simeu, N. and S. Mitra, *Disability and Household Economic Wellbeing: Evidence from Indonesian Longitudinal Data.* Oxford Development Studies, 2019. **47**(3): p. 275-88.

63.	Solmi, F., M. Melnychuk, and S. Morris, *The cost of mental and physical health disability in childhood and adolescence to families in the UK: findings from a repeated cross-sectional survey using propensity score matching.* BMJ Open, 2018. **8**(2): p. e018729.

64.	Touchet, A. and M. Morciano, *The disability price tag: technical report*. 2018, SCOPE: London, UK.

65.	Van der Heyden, J., et al., *Activity limitations predict health care expenditures in the general population in Belgium.* BMC Public Health, 2015. **15**: p. 267.

66.	Vu, B., et al., *The costs of disability in Australia: a hybrid panel-data examination.* Health Economics Review, 2020. **10**(1): p. 6.

67.	Wang, X., *Economic costs and health burden of vision problems in Singapore* 2016.

68.	Whitney, D., et al., *Economic burden of paediatric-onset disabilities among young and middle-aged adults in the USA: a cohort study of privately insured beneficiaries.* BMJ Open, 2019. **9**(9): p. e030490.

69.	Whitney, D.G., et al., *Prevalence of high-burden medical conditions and health care resource utilization and costs among adults with cerebral palsy.* Clinical Epidemiology, 2019. **11**: p. 469-481.

70.	Wilkinson-Meyers, L., et al., *To live an ordinary life: Resource needs and additional costs for people with a physical impairment.* Disability & Society, 2015. **30**(7): p. 976-990.

71.	Wu, J., et al., *Health care resource use and direct medical costs for patients with schizophrenia in Tianjin, People's Republic of China.* Neuropsychiatric Disease and Treatment Vol 11 2015, ArtID 983-990, 2015. **11**.

72.	Xiang, X.L. and R.P. An, *The Impact of Cognitive Impairment and Comorbid Depression on Disability, Health Care Utilization, and Costs.* Psychiatric Services, 2015. **66**(11): p. 1245-1248.

73.	Zan, H. and R.L. Scharff, *The heterogeneity in financial and time burden of caregiving to children with chronic conditions.* Maternal and Child Health Journal, 2015. **19**(3): p. 615-625.

74.	Zhang, H., et al., *Direct medical costs for patients with schizophrenia: A 4-year cohort study from health insurance claims data in Guangzhou City, Southern China.* International Journal of Mental Health Systems Vol 12 2018, ArtID 72, 2018. **12**.
